# Assessment of radiocesium contamination in frogs 18 months after the Fukushima Daiichi nuclear disaster

**DOI:** 10.1038/srep09712

**Published:** 2015-04-10

**Authors:** Noe Matsushima, Sadao Ihara, Minoru Takase, Toshihiro Horiguchi

**Affiliations:** 1 Research Center for Environmental Risk, National Institute for Environmental Studies, 16-2 Onogawa, Tsukuba, Ibaraki, 305-8506, Japan; 2 Environmental education, regional education development, Hokkaido University of Education, 1-15-55 Shiroyama, Kushiro, Hokkaido 085-8580, Japan; 3 Institute for Amphibian Biology, Graduate School of Science, Hiroshima University, Higashi-Hiroshima, Hiroshima 739-8526, Japan; 4 Research Center for Environmental Risk, National Institute for Environmental Studies, 16-2 Onogawa, Tsukuba, Ibaraki 305-8506, Japan

## Abstract

We investigated the accumulation of radionuclides in frogs inhabiting radioactively contaminated areas around Fukushima Daiichi Nuclear Power Plant (FDNPP) to search for possible adverse effects due to radionuclides. We collected 5 frog species and soil samples in areas within and outside a 20-km radius from FDNPP in August and September 2012 and determined their radiocesium concentrations (^134^Cs and ^137^Cs). There was a positive correlation between radiocesium concentrations in the soil samples and frogs, and the highest concentration in frogs was 47,278.53 Bq/kg-wet. Although we conducted a histological examination of frog ovaries and testes by light microscopy to detect possible effects of radionuclides on the morphology of germ cells, there were no clear abnormalities in the gonadal tissues of frogs collected from sites with different contamination levels.

The Fukushima Daiichi Nuclear Power Plant (FDNPP) was severely damaged by the Great East Japan Earthquake and Tsunami of 11 March 2011, releasing large amounts of radionuclides over a wide area^1^,^2^. After the radioactive fallout from the FDNPP, the wildlife in these areas was exposed externally and internally to radioactive contaminants.

Reports about the radioactive contamination and radiation effects on animals and plants in Fukushima are gradually increasing. The Fukushima prefectural government and the Ministry of the Environment are monitoring the accumulation of radionuclides in the wildlife after the FDNPP accident, and most of the examined species are popular for hunting and fishing^3^,^4^. Therefore, there is little information about the accumulation of radionuclides in species that are unsuitable for consumption^5^,^6^,^7^,^8^.

After severe nuclear accidents such as the Chernobyl accident, the biological impact of radioactive exposure has been investigated, and several studies have reported on the radiation damage to wildlife^9^,^10^. There are now a few studies about the biological impacts of radiation in contaminated areas in Fukushima, and they have reported aberrations in insects^11^,^12^ and DNA damage in earthworms^13^, but no radiation effects on bull testes^14^ or wild boar DNA^13^. There seems to be a variation in the radioactive sensitivity among species, suggesting that it is difficult to predict the consequences of radioactive exposure on species and ecosystem services^15^. To assess the radioactive impact on wildlife and ecosystems in the contaminated area around the FDNPP, much research on the radioactivity accumulation and health conditions of wildlife is needed.

In this study, we investigated the accumulation of radioactive contamination in frogs and its possible adverse effects. In Japan, most frog species inhabit rice paddy fields, and they are important organisms in paddy ecosystems. The areas around the FDNPP include many rice paddy fields. Frogs live close to the ground and they have a small home range. They are thin skinned and they absorb water through their skin. Therefore, they are constantly exposed to radionuclides in the environment both internally and externally^16^. Hence, it is biologically and ecologically important to elucidate the adverse effects of radionuclides on wild frogs.

We collected frogs and soil samples at survey sites in the highly contaminated area within a 20-km radius from the FDNPP and a neighbouring area in August and September 2012, and determined radiocesium concentrations in the frogs and soil samples. We conducted histological examinations of the ovaries and testes of the frogs caught at the survey sites by using light microscopy to search for aberrations in the morphology of germ cells, because we were more concerned about the impact of radioactivity on the individual frog's reproductive ability rather than on its survival ability. This is a preliminary analysis of the effects of radionuclides on the reproduction of frogs in wild populations.

## Results

### Frogs collected at surveys

**Figure 1:**
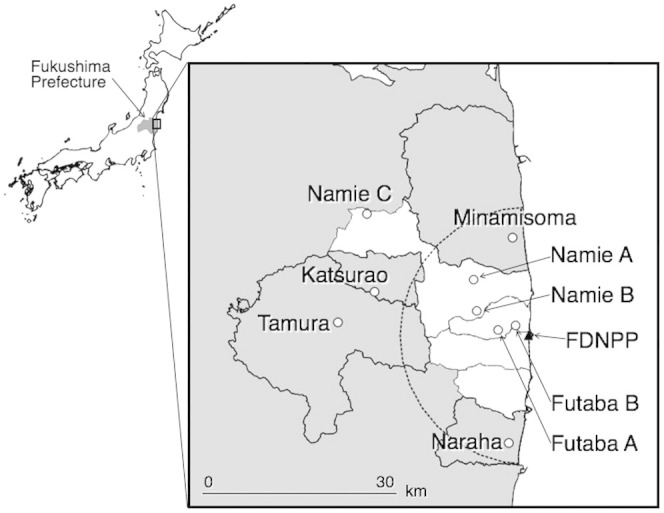
Map showing 9 survey sites around the Fukushima Daiichi Nuclear Power Plants (FDNPP). The white area in Fukushima Prefecture indicates the prohibited entry area due to the risk of radiation exposure in 2012. The dotted line indicates a radius of 20 km from the FDNPP. This map was illustrated with the open source software QGIS (http://www.qgis.org) (Administrative zones data sourced National Land numerical information from Ministry of Land, Infrastructure, Transport and Tourism).

We collected 135 live individuals of 5 species at 9 sites (*Rana japonica*, *R. ornativentris*, *R. tagoi tagoi*, *Pelophylax porosus porosus,* and *Hyla japonica*; Table 1 and Fig. 1). Although we could not catch frogs at several sites, we determined the presence of some species by sightings and hearing their calls during surveys. In the August survey, we could not catch *P. porosus porosus*, although we found it at Naraha. We heard calls of *R. catesbeiana* at Futaba B and found a larva of *Rhacophorus arboreus* at Namie C. In the September survey, we heard calls of *H. japonica* at Namie A, Namie B, Futaba A, Futaba B, and Tamura.

### Radiocesium concentrations of frogs and soils

From the total 135 collected frogs, 125 were used for the determination of radionuclides (mainly radiocesium). We detected radiocesium in 21 composite samples of *R. japonica*, 2 composite samples of *P. porosus porosus*, 2 composite samples of *H. japonica*, 1 composite sample of *R. ornativentris*, and 1 composite sample of *R. tagoi tagoi* (Table 2). Ten animals were not used for the determination of radiocesium because their body size was too small to meet the minimum requirements for determining radiocesium contents.

A female *R. japonica* at Minamisoma showed the lowest concentration of radiocesium (the sum of ^134^Cs and ^137^Cs) (36.81 Bq/kg, snot-vent length (SVL) = 62.68 mm), and a composite sample of 5 males and 3 juveniles (sex unknown) of *R. tagoi tagoi* at Namie C showed the highest concentration of radiocesium (47,278.53 Bq/kg, mean SVL of males = 37.65 mm, mean SVL of juveniles = 11.17 mm).

**Figure 2:**
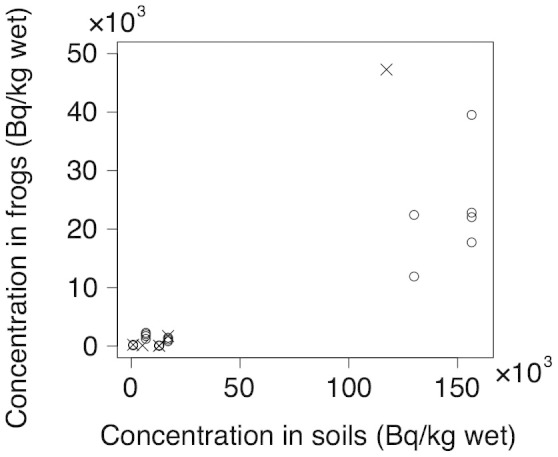
Radiocesium concentrations (the sum of ^134^Cs and ^137^Cs) of frogs and soil samples at each site. Open circles and cross marks indicate *Rana japonica* and other species, respectively.

The radiocesium concentrations in all species of frogs were positively correlated with those in soil samples (Spearman's rank correlation: *r* = 0.591, P = 0.001, n = 27, Fig. 2). We could not compare the radiocesium concentrations of frogs between species because we could get only a few animals of most species. In *R. japonica*, for which we could get animals at most survey sites, the radiocesium concentrations in frogs were correlated with those in the soil (*r* = 0.591, P = 0.005, n = 21).

**Figure 3:**
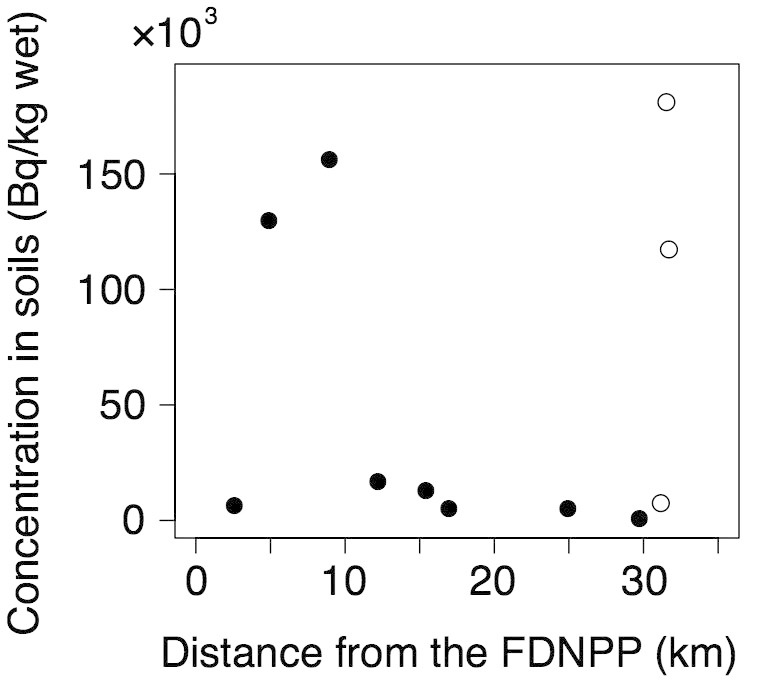
Radiocesium concentrations (the sum of ^134^Cs and ^137^Cs) of soil samples at each site and distance from the FDNPP. Open circles indicate soil samples collected at Namie C. Namie C had 3 soil samples because this site had 3 breeding pools within 840 m.

Radiocesium concentrations in soil samples were correlated with the air radiation dose rates at sites (*r* = 0.773, P = 0.008, n = 11), but not with the distance from the FDNPP to each site (*r* = −0.018, P = 0.968, n = 11, Fig. 3). Since the mean activity ratios of ^134^Cs/^137^Cs (decay correlated to 11 March 2011) were 1.062 ± 0.074 SD for frogs and 0.997 ± 0.029 SD for soil samples, and these ^134^Cs/^137^Cs ratios were nearly equivalent to that of the FDNPP fallout^17^, we regarded the radiocesium in samples as derived from the FDNPP fallout.

### Histological morphologies of ovaries and testes of the frogs

Of 29 males, 3 males of *R. japonica* showed testis-ova. One each of these males was caught at Futaba A, Futaba B, and Minamisoma in the August survey. Of 40 females, 13 females of *R. japonica* showed densely-stained cells (DSCs), which looked like degenerative oocytes, in the ovary. It is difficult to statistically confirm the difference in frequencies of females with DSCs between sites with different levels of radioactive contamination owing to the small numbers of animals. Of 28 females caught in the August survey and 12 females caught in the September survey, 6 and 7 females, respectively, showed DSCs. The frequency of females with DSCs in the September survey was significantly greater than that in the August survey (Fisher's exact test; P = 0.02). Females with DSCs were likely mature according to their body size^18^ (ranges of SVL, females with DSCs: 33.31–37.00 mm, females without DSCs: 21.93–62.57 mm). Therefore, although it is unclear what DSCs biologically or physiologically mean in ovaries, it would appear that DSCs are often found in ovaries of mature female *R. japonica*. Of 7 females of *H. japonica*, 6 females showed DSCs, and these were caught in the less contaminated sites of Minamisoma and Naraha.

## Discussion

In this study, we found that the radiocesium concentrations were rather high in frogs caught within a 20-km radius of the FDNPP. The concentrations found these frogs were positively correlated to radiocesium concentrations of the soil at the survey sites. Since there are very few studies of radioactive contamination of amphibians in Fukushima, our study is an important report about high radioactive contamination in frogs captured within a short period after the accident. The Ministry of the Environment reported radiocesium concentrations in some frog species within a 20-km radius of the FDNPP in May 2012, and high concentrations of radiocesium in four frog species (39,000–52,000 Bq/kg-wet at an air dose rate of 11.3–21.6 μSv/h) were found^19^. Additionally, monitoring data on the radiocesium concentrations of some amphibian species caught at lakes and rivers around the 20-km radius area in July and August 2012 reported low concentrations of radiocesium (68–750 Bq/kg - wet) in frogs and tadpoles caught in these low contaminated areas^5^. This indicates that radioactive element accumulation in frogs is influenced by radiocesium concentrations in their habitats, and that frogs did not migrate between sites with different levels of radioactive contamination. On the other hand, the Ministry of the Environment reported a markedly contaminated frog (*Buergeria buergeri* 160,000 Bq/kg-wet at 11.3 μSv/h) caught at a certain location in Minamisoma. *B. buergeri* inhabits forests. Similarly, *Rana tagoi tagoi,* which showed the highest concentration of radiocesium in our surveys, inhabit forests although the lives of both species are ecologically different. However, it is difficult to indicate the relationship of contamination levels among frog species and the environment of habitats because only a small number of samples were investigated in the present study.

In the present study, we did not observe clear aberrations in the morphology of germ cells in the testes and ovaries of frogs caught in the contaminated sites; however, not all aberrations may have been visible to us. The presence of testis-ova in male *R. japonica* was independent of contamination levels. In wild populations in several sites of Japan, however, male frogs with testis-ova were found frequently before the FDNPP accident. Male frogs of six species with testis-ova were observed at the ratio of 0% to 62.7% in Japan^20^,^21^, depending on their species, and the ratio of male *R. japonica* with testis-ova was 11.1%^20^. In this preliminary survey, meanwhile, we could not identify any histological abnormalities of the ovaries of *R. japonica*, nor were there clear differences in the morphologies (i.e. size and colour) and germ cells of the ovaries of females among survey sites. There is very little information about the radiation effects on frogs in wild populations. For example, in the investigations that were carried out both in the year when the Chernobyl accident happened and 3–8 years after the accident, chromosome aberrations and aberrant cells in bone marrow were observed in *R. temporaria* L. in contaminated regions in Belarus^10^. However, no details are known about radiation damage to frogs at the cellular level, or the consequences of long-term exposure to radiation at individual levels. Therefore, it is important to continue to investigate the radiation effects on wildlife.

Most of these frogs in Namie A, where we caught the most *R. japonica*, appeared to have been born in the spring of 2012 based on their body size. In April 2013, we found egg masses of *R. japonica* and *R. ornativentris* in survey sites within the contaminated area. These observations indicate that frogs could oviposit their eggs there in 2012 and 2013. Therefore, internal and external exposure to radioactive materials does not seem to have severely affected the reproductive behaviour or the maturation of eggs and sperm in frogs. As *R. japonica* has a lifespan of several years^18^, it is possible that the radioactive materials had little damage on the reproductive abilities of frogs that were born before the accident.

On the other hand, the effects of radiation (possibly, low-dose radiation) on the early developmental stages of frogs (e.g. eggs and tadpoles) were unknown in this study. In some laboratory experiments that were performed with high dose irradiation, sensitivities of zygotes and hatchings to radiation were higher than that in juvenile and adult frogs^22^,^23^ exposed to 956 rad of X-rays^22^ and gamma radiation of 0.3 Gy min^−1^ from ^60^Cobalt and 0.05–0.26 Gy h^−1^ from ^137^Cs^23^. Chromosome aberrations and a decrease in the number of normally metamorphosed frogs were observed in frogs produced from gametes exposed to 150–350 rad of X-rays and 100–250 rad of neutrons^24^. In laboratory experiments on other amphibians, low dose irradiation did not influence body size, age at metamorphosis^25^, or juvenile survival rates^26^ when spadefoot toad tadpoles and salamander embryos were reared under low dose rates of gamma irradiation (up to 222 mGy d^−1^ and 490 μGy h^−1^ (corresponding to 11.76 mGy d^−1^), respectively). However, these studies did not examine the effects of internal exposure to radioactive materials. Both tadpoles and adults in contaminated areas always assimilate radiocesium in the natural environment^27^. It is necessary to investigate not only the effects of external exposure but also those of internal exposure on eggs and tadpoles and to continuously monitor frog reproduction in highly contaminated areas.

We caught common species of frogs in our survey area and found the species on which our surveys about biota and the environment in the eastern area of Fukushima prefecture would be based^28^,^29^,^30^. In the eastern area of Fukushima prefecture, five frog species inhabit paddy fields and lowlands (*R. japonica*, *H. japonica*, *P. porosus porosus*, *Glandirana rugosa*, and *Rhacophorus schlegelii*)^30^. Generally, most frogs that breed in paddy fields are frequently found in paddy fields after their breeding season. Of five species, *R. schlegelii* uses paddy fields as reproductive sites in early summer and live in forests in other seasons. *G. rugosa* is an endangered species in the Fukushima prefecture^31^. Therefore, we had expected to get more frogs of three common species (*R. japonica*, *H. japonica*, and *P. porosus porosus*). Unexpectedly, however, we found only a small number of frogs at most survey sites. One possible reason is that it rained less than usual in the summer of 2012. Therefore, most of the frogs might have left for moist environments since the complete evacuation of residents from the area meant that there was no water body in the paddy fields. However, we caught many frogs at Minamisoma, where water was supplied from a spring and grooves. Therefore, this site was a suitable wet environment for inhabitation and breeding of frogs.

Another reason is that most paddy fields had changed into grasslands without pools of water. There were many paddy fields in these areas. Every year, rice producers irrigated rice fields to cultivate rice from the spring through the summer. Therefore, paddy fields had been used by amphibians as breeding pools in place of wetlands. Since all residents in the radioactively contaminated area were immediately evacuated after the FDNPP accident, no agricultural activity had been conducted in the area and neighbouring areas since the accident, and consequently, most paddy fields have dried and changed into grasslands. For many amphibians, the aridification of paddy fields means a rapid decrease in suitable environments. The same is also true for many other organisms of paddy ecosystems, such as aquatic insects and fishes. We should pay attention to rapid changes in environments caused by no human activity due to exhaustive escape from the accident and consider the biodiversity conservation in radioactively contaminated areas as well as radiation impacts on wildlife health.

## Methods

### Study sites and sample collection

We surveyed and caught frogs at 5 sites within the 20-km radius area and at 4 sites surrounding this area (Fig. 1, Table 1). Entry into the area is still prohibited as an evacuation zone (The area was reorganized into the ‘difficult-to-return zone’ in 2013) because of the risk of radiation exposure.

The surveys in restricted area were always performed by 3 people. Two people walked around a site, searching for and catching frogs for thirty minutes, while the other was in charge of safety management and helping the researchers take off protective clothing. When frogs were not caught but they were observed or their calls heard, we recorded their species. These surveys were conducted twice in 2012 (27–28 August and 20–21 September). In August, we did not conduct the survey at Tamura, Katsurao, or Namie C.

Except for Katsurao and Namie C, paddy fields were being used as survey sites before the accident. In Namie C and Katsurao, there were pools surrounded by forests and there were paddy fields used before the accident within 500 m from these sites. When we surveyed, in Naraha and Tamura sites, there were experimental paddy fields created to check for radioactive contamination in rice. Other paddy fields had pools or marsh in grasslands, although most paddy fields there dried to change into grasslands owing to lack of agricultural activity in the evacuated area. Thus, these survey sites were selected as the place where frogs may be able to inhabit.

To investigate the radioactive contamination in the frog habitat, radiation dose rates at 1 m above the ground surface were applied and measured by NaI scintillation survey meter (Aloka TCS-161) and CsI scintillator (HORIBA PA-1000 Radi). For the determination of radionuclides in soil, soil samples were collected from the surface where there was very less vegetation (i.e., one soil sample per site). At Namie C, there were 3 small pools within 840 m and we observed eggs of *R. ornativentris* in all pools in March 2012. Therefore, although we collected surface soil from beside each pool to take account of the differences in contamination between pools, all frogs collected around pools were combined because we could collect only a few frogs there (see Table 2).

We collected frogs irrespective of the body size and age. No tadpoles were collected, neither for histological examination nor for determination of radiocesium levels. All live frogs caught were transferred to a laboratory in Tsukuba and kept alive in a room maintained at 20°C until dissection of the gonads. Under deep anaesthesia with diethyl ether, live frogs were measured (snout-vent length) and weighed, and the gonads were immediately dissected for histological examination. All tissues removed for dissection were preserved in 70% ethanol after fixation in Bouin's solution for 48 h and then embedded in paraffin. They were cut into 5–6 μm sections using a microtome and subsequently stained with hematoxylin and eosin. The experiments were carried out in accordance with the approved guidelines prepared by National Institute for Environmental Studies, Japan.

### Data analysis

Radiocesium (^134^Cs and ^137^Cs) concentrations of frogs and soil samples were analysed using a gamma spectrometer with a germanium semiconductor detector (GMX45P-76, Seiko EG&G Ortec, Tokyo, Japan). Gamma-ray emissions were measured at energies of 604.7 keV (^134^Cs) and 661.6 keV (^137^Cs). Detection time was 203–40,000 s and 26–995 s for frogs and soil samples, respectively. All soil samples and the tissues of frogs were maintained at 4°C and −20°C, respectively, until measurement. Whole bodies of individuals whose gonads maintained removed and individuals that died before dissection were used for the determination of radiocesium. Frogs were separated according to body size and sex for determination of radiocesium. Large frogs were measured alone. To meet the minimum requirements for determining radiocesium contents, small frogs were collected and measured as composite samples (Table 2). Some composite samples contained frogs of both sexes or whose sex was unknown.

Spearman's rank test was used to assess the relationship between radiocesium concentrations of frogs and soil samples from the survey sites from where the frogs were captured. To predict contamination levels easily, we also found correlations between the radiocesium concentration in soil samples and air radiation dose rates at each survey site, and between the radiocesium concentration in soil samples and distance from FDNPP to each respective survey site. Statistical analyses were performed using the statistical software R ver. 3.0.2 (R Development Core Team 2013).

## Author Contributions

N.M., S.I. and T.H. designed the study and collected the samples. M.T. analysed all sections of gonadal tissues of frogs. N.M. analysed all of the data and wrote the manuscript text with information from all the authors.

## Figures and Tables

**Table 1:** The numbers and species of frogs caught and air radiation dose rates and soil radiocesium concentrations at each site. Air radiation dose rates were measured on 20–21 September 2012. Upper and lower numbers indicate the numbers of frogs collected in August and September, respectively. The numbers in parenthesis indicate the numbers of frogs whose gonads were observed

Sites (Elevation)	Air radiation dose rate (μGy/h)§	Radiocesium concentration in soil samples (Bq/kg)	*Rana japonica*	*Pelophylax porosus porosus*	*Hyla japonica*	*R. ornativentris*	*R. tagoi tagoi*
Restricted area							
Namie A (48 m)	5.89	16,928.373	28 (20)	2 (2)	0	0	0
			0*	0	0**	0	0
Namie B (84.7 m)	35.13†	156,440.100	9 (7)	0	0	0	0
			3 (3)	0	0**	0	0
Namie C (539–556 m)	19.75†	7,490.959	-	-	-	-	-
	24.00†	181,075.630	0	0	0	2(1)	8(5)
	15.75†	117,311.560					
Futaba A (43 m)	64.00†	129,965.440	3 (3)	0	0	0	0
			2 (1)	0	0**	0	0
Futaba B (43.3 m)	3.04	6,721.352	11 (11)	0	1 (1)	0	0
			7 (7)	0*	0*	0	0
Other area							
Minamisoma (1.1 m)	0.31	12,824.942	14 (8)	0*	3 (3)	0	0
			5 (5)	2 (1)	7 (4)	0	0
Tamura (562.1 m)	0.78	861.924	--	-	-	-	-
			2 (2)	0*	0**	3 (2)	0
Katsurao (569.7 m)	1.37	5,383.595	--	-	-	-	-
			1 (1)	0	0	1 (1)	2 (2)
Naraha (7.9 m)	1.72	5,181.585	1 (1)	0*	18 (4)	0	0
			0	0	0	0	0

§Air radiation dose rates were converted from μSv/h to Gy/h according to the Guide for Environmental Radiation Monitoring established by the Nuclear Safety Commission of Japan in 2008 (http://www.nsr.go.jp/archive/nsc/anzen/sonota/houkoku/houkoku20080327.pdf, in Japanese).

†indicates that the air radiation dose rate was measured by CsI scintillator (HORIBA PA-1000 Radi).

*Frogs that were not caught although they were observed.

**Frogs that were not caught although their calls were heard.

**Table 2:** Radiocesium concentrations in frogs at each survey site. The n and mean SVL respectively indicate the numbers and mean snout-vent length of frogs included in composite samples. F, M and U indicate female, male and unknown sex, respectively and numbers indicate the number of individual sex in each composite sample. A and S indicates August Surveys and September Surveys respectively, and numbers indicate the number of collected frogs in each survey

Site	Radiocesium concentration in frogs (Bq/kg)	n	Mean SVL (SD) (mm)	Sex	Collected date
*Rana japonica*					
Namie A	821.76	11	24.95 (2.97)	F:10, U:1	A
Namie A	1,404.69	12	23.09 (2.70)	F:1, M:10, U:1	A
Namie A	1,415.68	3	43.25 (5.43)	M	A
Namie A	1,099.69	2	41.60 (1.65)	F:1, M:1	A
Namie B	22,039.70	6	36.87 (5.17)	F:4, M:2	A
Namie B	22,813.36	1	62.57	F	A
Namie B	39,531.67	3	50.05 (4.55)	M	A:2, S:1
Namie B	17,729.06	2	49.49 (0.30)	F	S
Futaba A	11,892.53	3	41.99 (1.28)	F	A
Futaba A	22,420.58	2	43.09 (2.00)	M	A
Futaba B	2,273.88	7	35.81 (4.20)	F:6, M:1	A
Futaba B	1,706.45	4	40.05 (2.99)	M	A
Futaba B	2,014.85	2	40.57 (4.72)	M	S
Futaba B	1,223.79	5	35.91 (2.72)	F	S
Minamisoma	75.76	6	34.71 (5.64)	F	A:5, S:1
Minamisoma	65.79	5	35.99 (5.31)	M	A
Minamisoma	81.01	4	45.01 (2.69)	F	A:3, S:1
Minamisoma	36.81	1	62.68	F	A
Minamisoma	74.53	3	40.50 (5.38)	M	S
Tamura	228.97	1	61.91	F	S
Tamura	104.50	1	52.07	M	S
*Pelophylax porosus porosus*					
Namie A	1,707.08	1	58.64	M	A
Minamisoma	43.15	2	41.36 (4.77)	F:1, M:1	S
*Hyla japonica*					
Minamisoma	46.19	10	27.16 (3.67)	F:6, M:4	A:3, S:7
Naraha	106.44	17	15.93 (1.98)	F:2, M:5, U:10	A
*R. ornativentris*					
Tamura	220.64	3	38.21 (6.23)	F:1, M:1, U:1	S
*R. tagoi tagoi*					
Namie C	47,278.53	8	27.72 (14.39)	M:5, U:3	S
